# More on responsibility of psychiatrists in Indian setting

**DOI:** 10.4103/0019-5545.37676

**Published:** 2007

**Authors:** Prakash Gangdev

**Affiliations:** Department of Psychiatry, University of Western Ontario, Canada. E-mail: prakashgangdev@sjhc.london.on.ca

Sir,

This timely editorial on the responsibilities of psychiatrists[1] clarifies as well as raises a number of issues. In the Indian context, advocacy, confidentiality and projection of psychiatric patients/problems in news and entertainment media are of particular relevance. Of more importance is the role of research community to enhance research and dissemination of knowledge.

Recently, an ex-movie star, relatively young, who had suffered from schizophrenia, died in isolation in Mumbai. Her failing mental and physical health went unrecognized and untreated, and it appears that, barring some media reports, there was no formal reaction or attempt to analyze the meaning and implications of her death. Despite her wealth and status, she did not receive care/treatment in keeping with her needs. This raises the question of fate of the unknown number of patients with no such status/wealth at their disposal. Whether this worries the nation or there are resources and ‘operational’ legal frameworks that cater to the needs of such patients remains unclear. While it is a matter of pride to see medical tourism being promoted and flaunted, the provision of care to the very vulnerable patients remains far from satisfactory.

Pejorative terms are sometimes used, and there appear to be no guidelines for the media in this regard. In this context, the movie star was badly criticized for her symptoms (which included accusations against other actors); I remember a televised interview wherein (I suspect) a professional was assisting in eliciting the symptoms for public consumption. This certainly calls for some self-regulation. Perhaps, the IPS and its other affiliated bodies should monitor the nature and quality of reporting and set up educational forum for journalists/editors.

Regarding depiction of mental illness in movies, the less said the better. In one movie, a character with mental disorder is made to marry as part of treating his mental illness, which, it was suggested, was caused by celibacy, and if he were not married soon his ‘brain nerves’ would burst. Most often, the story writers/directors/actors do not seem to distinguish between intellectual disability and psychotic disorder and end up with bizarre depictions, like the patient making strange body movements. Sometimes, the psychiatrists are buffoonish and end up falling in love with patients, breaching confidentiality, prescribing ECT in a sinister manner and offering weird advice. Most of the filmmakers have commercial interest and do not worry about clinical accuracy and contemporary treatment principles. The IPS should object to any out-of-line depiction of the patients or profession.

While advancements have been made in various spheres in India, including the medical profession, literature in psychiatry appears to be less, in that the IJP is published only four times a year even in its 48^th^ volume, and is not yet an indexed journal - that should not matter. However, this is surprising as there are very many esteemed and internationally recognized colleagues in the country who frequently write for international journals. To enhance the profile of Indian psychiatry locally and internationally, we would appreciate if they publish their research in the IJP, and that the journal were to be published monthly. It will be timely that India is emerging as a hub for medical research. Ultimately, the journal is made successful by loyal readership and the impact it has on their practice and not by being indexed.
